# Several Glimpses, Mostly Medical, of Lands across the Seas1Read before the Obstetrical Section, Buffalo Academy of Medicine, September 25, 1900.

**Published:** 1900-11

**Authors:** Francis Eustace Fronczak

**Affiliations:** Buffalo, N.Y., 508 Fillmore Avenue


### SEVERAL GLIMPSES, MOSTLY MEDICAL, OF LANDS ACROSS THE SEAS.[1]

By FRANCIS EUSTACE FRONCZAK, A.M., M. D., Buffalo, N. Y.

DR. ISAAC WATTS, the famous English author, wrote on the advantages of traveling as follows: "Nothing tends so much to enlarge the mind as traveling, that is making a visit to other towns, cities, or countries, besides those in which we were born and educated."

On the invitation of the chairman of the Obstetrical Section, I am expected to tell you tonight a few interesting things which I saw in my recent travels in lands across the seas. These things were not necessarily to be confined to exclusively medical topics, I believe, and yet I think I can eject enough, relating to our profession to warrant the chairman in inviting, and me in appearing before you.

Before proceeding further I wish to announce if anyone here present has a patient whom he desires to send to a temperate, equable, but humid climate, that the Azores is the place. The islands, situated as they are in the middle of the Atlantic, are ever moist, in fact, the humidity is so great there, that paper hangings will not adhere to the walls, and the veneering of furniture strips off. In one word, it is a case of chronic hyperidrosis with everything and everybody there, which even atropin and agaricin will not help. But let me proceed.

We know that the Arabians played a very important part in making the history of medicine. In fact, from the seventh until the fifteenth century, Arabians were the leaders in the study of our science. The Pyrenean peninsula was one of their strongholds, and even now, almost 500 years after their downfall in Spain, we find Arabs and Moors in all their oriental glory, with many of their ancient customs and traditions preserved in the Don Kingdom. These men delight in "doing" people, especially the foreigners—meaning the visitors to Spain--by two means, either by selling to them a worthless thing for a high price, and appearing in the capacity of merchants, or by presenting a person with a charm, which will protect him from all physical evil and disease. For this charm, if one keeps it, he has to give a suitable money gift, or the charm will not work.

Almost the entire Moorish population in Gibraltar, the principal, and now about the only place in Europe where they live, is engaged in this simple medical pursuit. These men are dirty, half naked, and

1. Read before the Obstetrical Section, Buffalo Academy of Medicine, September 25, 1900.

they emit a peculiar specific, pungent odor. This odor has been mentioned by other travelers, but as yet no one explains the cause. It is possible that the asafetida-like aroma of a peculiar looking fruit, or onion, which they eat in great abundance, mixed with the glandular secretions and dirt, all undergoing a change, produces this nauseating smell. But not only the Moors in Spain are a little behind in their hygienic studies and practice. Our late opponents in the little diversion which we had in the Antilles, and near Manila, about two years ago, have the same weakness. By the last census in 1896, there were about 19,000,000 people living in Spain, of whom about 31,000 were medical doctors, that is 613: 1. Besides, there are about the same number of men and women practising who have had no medical studies. The death-rate of Spain, according to recent reports is 32 per 1,000, while the mortality in the United States is about 18.6 per mil. The climate of Spain seems to be delightful and healthy, and we do not hear of any great epidemics there. Why, then, the great mortality? By far the greater number of deaths must be due to lack of even elementary hygienic arrangements in villages and cities. The physicians have not struck me as being progressive, and I have heard time and again from English physicians there, that many Spanish surgeons smoke cigarettes (all Spaniards, men and women, are inveterate smokers) even during operations, and that they will make an incision, take a puff of their smoking weed, and without washing their hands, proceed with the operation. I do not mean to say that all Spanish surgeons do that, but that a good many do it. These things were mentioned in Gibraltar, Malaga and Granada, and the physicians, on the whole, do not enjoy good reputation in Spain, and yet, that country has many progressive men who desire to hold high the standard of medical knowledge. The proof of which, may be the fact, that the $1,000 prize established at the 12th International Medical Congress, at Moscow, in 1897, for medical work of the greatest benefit to humanity, has been awarded to Professor Ramon y Cajal, of the University of Madrid, for his researches on the minute structure of the nervous system.

I believe I saw more goitre in Spain than in any other part of the world, especially in the mountain districts, as in the Sierra Nevada range, and I noticed that quite a percentage of people had large appenditures at the throat. I made inquiry as to the cause of it, and water was made the scapegoat for the goitre. I do not know whether this is so or not, but some authors mention water as an etiological fact of this enlargement of the thyroid, especially the water of the Alps of Switzerland and the Apennines in Italy; in fact, water in every mountainous district is supposed to have the quality of producing this condition. I have been in the Alleghanies and Adirondacks, but have not noticed goitre so much on the American continent.

The medicines of Spanish apothecaries are made entirely different from those of other parts of Europe and our own country, and the drug stores are all very small. I believe it is advisable for an American traveling in the Pyrenean peninsula to be provided with a small traveling medicine chest.

Now let us cross to another continent just for a moment. It is but a few hours from Europe to Africa at the Strait of Gibraltar. And yet, in that short time, we seem to have been transferred to an entirely different world. If you care to see anything of oriental life, you need not go to Smyrna or Cairo. Less than two weeks journey from Buffalo will bring you to a place where you can have all of Eastern customs you want for some time. Just for a moment let me describe to you a town of several thousands of barbarians, dirty, ill-fed, half naked, and dwelling in abominable huts, and together with their beasts of burden occupying the only room, which is as large as a good-sized dry goods box. The streets being so narrow that hardly two people can pass each other, unpaved and uncleaned, ever smelling in the hot African sun, tend to produce nausea in the uninitiated. The filthiness of the streets, the sight of the barbarian people, ever yelling and quarreling, the braying of the asses, the unbearable odor of decaying matter, would without doubt be sufficient warranty for Dr. Wende, if he ran Tanjers, or Tetuan, or any of the cities of Northern Africa, to have them declared a nuisance, and have them razed to the ground. And yet the health of the coast cities of Mediterranean Africa is on the whole very satisfactory, thanks to the advantages of the climate, whereby the heat of the almost torrid sun is alleviated by the winds which blow from the ocean and the Mediterranean sea.

Morocco was at one time one of the leading educational countries of the world. Here lived and practised for a time the great physicians and fathers of medicine, Avicenna, Averroes and others, and as in other oriental countries, so also here, the priests are also a kind of medicine men. It is a great honor to be able to touch the mantle of a medical Moor, and a very highly prized privilege to be able to kiss the toe of a priest physician, for, by this grace, all diseases are banished from the fortunate man who has been permitted to do the dirty kissing act. How nice it would be if some of the honors which medical men possess among the Arabs, Moors and Beduins could be transferred here!

Let me make another jump; this time to Italy. The physicians there, especially in the southern part, seem to have only two agents in their pharmaceutical reservoir: castor oil and quinine. The first for everything for which the second is not used, and the second wherever it appears that a laxative cannot be used very well for any reason. The Roman fever, known in and about Rome, as the "febre aestivo-autumno," seems to be the scapegoat for most of the medical ignorance. There are many physicians in Italy, in fact too many, according to the complaints of doctors there, for Italians suffer as we in the United States of too many medical doctors in proportion to the necessities of the population.

There are twenty medical schools, colleges and universities in the country of the Cesars, having 6,386 students in the last academic year (1899-1900), running from 2009 in the University of Naples, to 15 in the free University of Ferrara. Notwithstanding the great complaints of the Italians, we are much worse than any other country in that respect, for we have six times as many physicians here as there are in Italy in proportion to the population, and on the same ratio and proportion, four times as many medical men as France, five times as many as Germany, and almost thirty times as many as Russia.

Here let me deviate a little from my tour. In 1899, there were only 18,334 physicians in the Russian Empire, though the population of the same was 128,932,173, while we have in America over 125,000 physicians, practising among about 75,000,000 inhabitants. And even though there are proportionately few medical doctors in Russia, and only six medical colleges, yet since January 1, 1900, to prevent any hyperproduction, a law has come into effect whereby in the first year of medicine no more than 100 students are to be admitted in the universities of Warsaw and Kazan, 200 in the University of Kieff and Charkow, and 250 in St. Petersburgh and Dorpath. This is truly a sensible, a wise, and I would say a humane law. For, is there a physician practising anywhere in this country, or even a layman, that is a non-professional man, who after due consideration will not admit and say that the 156 medical schools in the United States could be reasonably and with perfect safety for the profession, and for the country at large, be reduced to about 25? And that the 24,119 medical students who attended these 156 schools in 1899 are by far too many for the needs of the country. In 1898, there were graduated

5,725 men and women with the degree of Doctor of Medicine! Shades of Hippocrates and Galen! If this proportionate increase of medical doctors should continue much longer, where will they all make their living? Surely not by practising medicine. And similar conditions of medical men are also in Europe.

But enough of philosophising. Journey with me to Austro-Hungary. I will not detain you at Vienna, most of you have been there, studied there, or have read of the medical schools and the prominent medical men and of the Hapsburg capital's large and rich and well arranged hospitals. I will take you, for a moment, further north of the Empire. Cracow, in Galicia, formerly the capital of Poland, but a few miles from the Russian and German frontiers, has a very old university. In fact, a celebration of a quin-centennial jubilee took place in June last. The medical department, though by the bull of Pope Urban V., in 1364, was to have been organised at once, as the words of the act are plain in this respect: "Scholas debitas ex nunc ordinamus pro legendo jure canonico vel civili, *medicinis* vel artibus liberalibus," was not opened for a certainty until 1433, thus making it one of the oldest medical schools in the world, which have been continued unceasingly until the present time, teaching our science and progressing 'pari passu' with ideas of the various epochs and times. It was one of the humane schools from its very beginning, as one of its rules adapted early was, "that no distributor or giver of poisons, nor one who is an abortionist, whether by herbs, drugs or manipulations, can be a doctor of physic, under the pain of punishment by fire of hell." The school has now 67 teachers in various branches of medicine, about 350 students, 6 university clinics, each in a separate, classical, large modern building; one hospital, and nine institutions, in which are located the various scientific laboratories and museums. It was not my intention to lay so much stress on this university, but the fact, which I have mentioned, of its existence over five centuries, forced me to speak especially and to dwell a little longer on this medical school.

Now, another long jump, as I turn among my notes. Hotel Dieu, also Salpêtriere, in Paris, very large and well known hospitals, of which no doubt everyone has heard, are nothing else, in my humble opinion, than poor houses for the sick of Paris and neighboring country. I always believed that these institutions were something grand, up-to-date, modern hospitals. They are not; they are overcrowded, unclean, and do not, as a rule, impress a medical man with their antiquated arrangements. But enough; in closing my remarks,

I will say that these are but a few observations, taken at random, from my note-book and from the lingering vivid memories from lands across the sea.

508 FILLMORE AVENUE.

